# Improving the Bioaccessibility and Bioavailability of Carotenoids by Means of Nanostructured Delivery Systems: A Comprehensive Review

**DOI:** 10.3390/antiox11101931

**Published:** 2022-09-28

**Authors:** Camilla Molteni, Concettina La Motta, Fabio Valoppi

**Affiliations:** 1 Department of Pharmacy, University of Pisa, Via Bonanno 6, 56126 Pisa, Italy; 2 Interdepartmental Research Center Nutrafood “Nutraceuticals and Food for Health”, University of Pisa, Via del Borghetto 80, 56124 Pisa, Italy; 3 Department of Food and Nutrition, University of Helsinki, PL 66, Agnes Sjöbergin katu 2, 00014 Helsinki, Finland; 4 Faculty of Agriculture and Forestry, Helsinki Institute of Sustainability Science, University of Helsinki, 00014 Helsinki, Finland; 5 Department of Physics, University of Helsinki, PL 64, Gustaf Hällströmin katu 2, 00014 Helsinki, Finland

**Keywords:** nanotechnology, carotenoids, bioaccessibility, bioavailability, lipid-based nanocarriers, biopolymeric nanocarriers

## Abstract

Carotenoids are bioactive compounds provided by the diet playing a key role in maintaining human health. Therefore, they should be ingested daily in an adequate amount. However, even a varied and well-balanced diet does not guarantee an adequate intake, as both the bioaccessibility and bioavailability of the compounds significantly affect their absorption. This review summarizes the main results achieved in improving the bioaccessibility and bioavailability of carotenoids by means of nanostructured delivery systems, discussing in detail the available lipid-based and biopolymeric nanocarriers at present, with a focus on their formulation and functional efficiency. Although the toxicity profile of these innovative delivery systems is not fully understood, especially for long-term intake, these systems are an effective and valuable approach to increase the availability of compounds of nutritional interest.

## 1. Introduction

**Figure 1:**
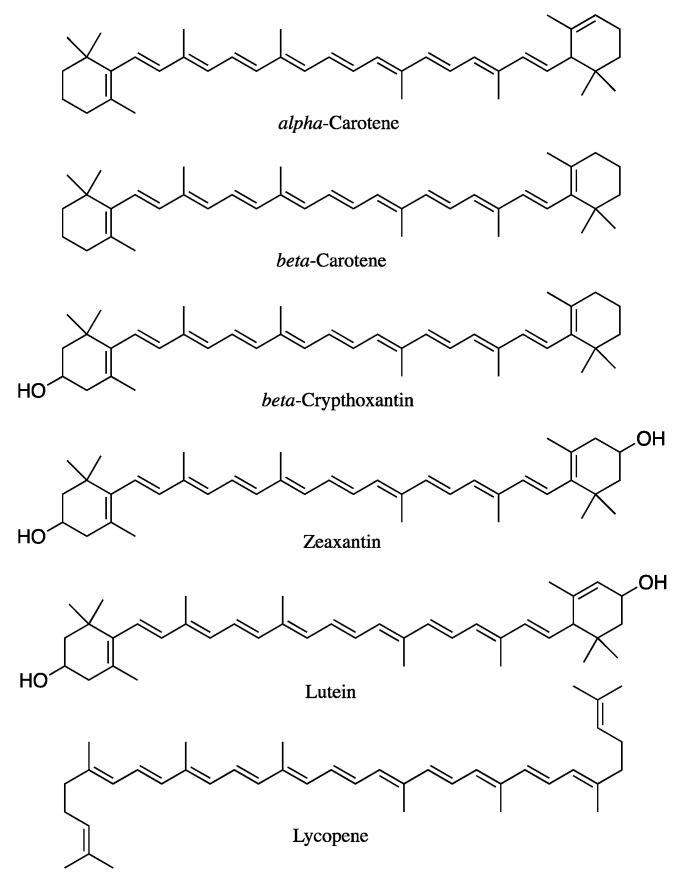
Chemical structures of relevant carotenoids.

Carotenoids are a broad group of naturally occurring tetraterpene pigments consisting of more than 1000 compounds [1], synthesized by plants, algae, some bacteria, fungi, and invertebrates. Carotenoids are not synthesized by mammals; therefore, their supply depends on dietary sources. Approximately 80–90% of carotenoid intake comes from fruit and vegetables, while the remaining 10–20% comes from animal products, such as milk, cheese, egg yolk, and butter [1]. Nearly 50 out of the 1117 different compounds identified to date are present in the human diet and can be absorbed by humans [2]. However, only α-carotene, β-carotene, β-cryptoxanthin, lycopene, lutein, and zeaxanthin (Figure 1) represent 95% of carotenoids found in human plasma and are associated with health benefits. Accordingly, these compounds are the most studied among the whole family [3]. Those bearing one or more β-ionone ring along their structure, such as α-carotene, β-carotene, and β-cryptoxanthin, have a key pro-vitamin-A role. Therefore, adequate intake is crucial to achieve sufficient levels of this micronutrient [4]. Moreover, thanks to their long, conjugated double-bond structure, all carotenoids possess antioxidant and radical-scavenging activities which, in turn, give the compounds key immune-stimulating properties and protective effects against cardiovascular diseases and UV exposure [5,6], thus making them a privileged class of derivatives endowed with nutritional interest.

## 2. Functional Relevance of Carotenoids

Several studies emphasized the effect of carotenoids on the immune response, pointing out their ability to increase the count of both CD4^+^ lymphocytes and lymphocytes expressing markers of cell activation, such as IL-2 and transferrin receptors. Furthermore, carotenoid supplementation was proved to enhance the long-term activity of natural-killer (NK) cells and suppress NF-kB activation, thus inducing anti-inflammatory effects [5]. At the same time, low levels of carotenoids, such as α-carotene, β-carotene, lutein, and zeaxanthin, were related to enhanced oxidative stress levels and inflammation, as well as high levels of IL-6 [7]. Moving from these premises, Iddir and co-workers [8] recently linked the ability of carotenoids to strengthen the immune system with the chance to fight severe infectious diseases, including the dreadful COVID-19, thus further highlighting the importance of an adequate intake of these compounds to cope with pathological conditions.

Carotenoids were also claimed to have protective effects on cardiovascular health. In particular, a negative correlation has been demonstrated between serum concentrations of β-carotene and the incidence of atherosclerosis, having high amounts of carotenoids that are protective against damage to vessel walls. According to Harari and co-workers [9] and Relevy and co-workers [10], the intake of both all-*trans*- and 9-*cis*-β-carotene reduces both the amount of plasma cholesterol levels and atherosclerotic lesions in apolipoprotein E-deficient mice. Moreover, Bechor, and co-workers [11] verified that 9-*cis*-β-carotene from the diet accumulates in peritoneal macrophages and increases cholesterol efflux to HDL, protecting against the development of atherosclerosis. As for the observed efficacy, Zhou and co-workers [12] brought into play the β-carotene oxygenase 1 (BCO1) activity, in charge of the conversion of the carotenoid to vitamin A, providing the evidence that this conversion regulates hepatic lipoprotein secretion and atherosclerosis development in mice. Amengual and co-workers [13] corroborated this evidence, demonstrating that the activity of BCO1 affects the total amount of circulating cholesterol both in mice and in young adults. Generally speaking, thanks to their radical-scavenging and antioxidant activities, carotenoids reduce the risk of cardiovascular diseases, including sudden cardiac death [14], some kinds of cancer, age-related macular degeneration [15], and also stroke and other causes of mortality, as recently demonstrated by a large prospective serological analysis conducted by Huang and co-workers [16] on almost thirty thousand men during 31 years of follow-up.

In addition, carotenoids demonstrated to decrease LDL-cholesterol plasma levels and improve insulin sensitivity and HDL efficiency, thus also playing a key role in reducing the risk of occurrence of metabolic syndrome [6,7,15].

An adequate intake of carotenoid is also crucial to support normal vision. Lutein, zeaxanthin, and meso-zeaxanthin are the main represented carotenoids in the visual-related tissues, being distributed within the macula and throughout the retina and brain visual cortex. Absorbing most of the incident light, they protect tissues from light-induced damage, also reducing light scattering and improving visual acuity. Their intrinsic anti-oxidant activity, also resulting in an anti-inflammatory efficacy, complements the functional relevance of these compounds in preventing the development of age-related diseases, such as macular degeneration and cataracts. Clinical and epidemiological studies also suggest their protective role against the development of diabetic retinopathy and glaucoma [17,18].

The presence of carotenoids in the skin, in the outer part of the stratum corneum, enhances the basal dermal defense against UV damage, thus promoting the skin’s health. In particular, a diet rich in lycopene and β-carotene proved to have protective effects towards sunburn and UV-induced erythema formation [19]. Several mechanisms were called into question to justify the observed outcomes, including the ability of carotenoids to prevent lipid peroxidation by quenching singlet oxygen or scavenging free radicals, to inhibit the UVA-induced expression of heme-oxygenase 1, involved in the regulation of cell proliferation, differentiation, and apoptosis, and to protect against mitochondrial DNA mutation related to UV-induced aging and carcinogenesis. In addition, carotenoids proved to inhibit metalloproteases, which are enzymes related to photo-aging, and counteract the immunosuppressive effects occurring as a consequence of excessive UV exposure [20].

A comprehensive revision of the literature also highlighted few studies seeming to suggest that carotenoid supplementation might have adverse effects, such as the CARET (β-carotene and retinol efficacy trial) [21], and the ATBC (α-tocopherol and β-carotene for cancer-prevention study) [22]. Both the investigations were designed to verify the ability of β-carotene to reduce the risk of lung cancer when administered in high doses to smokers, but were stopped ahead of schedule as β-carotene supplementation led to a statistically significant increase in lung cancer incidence and overall mortality compared to the placebo. However, Duffield-Lillico and Begg [23] justified the observed events by the doses of β-carotene used, resulting in supra-physiologic serum concentrations. Furthermore, similar studies on the preventive effects of β-carotene, such as the Physicians’ Health Study [24], Women’s Health Study [25], and Skin Cancer Prevention Study [26], did not show any adverse effects with respect to cardiovascular, cancer, and mortality endpoints. The same was also true for two additional reports concerning two single clinical cases of women displaying either maculopathy or retinopathy when supplemented with carotenoids, such as lutein [27] and canthaxanthin [28], respectively. Additionally, in these cases, both the patients experienced high, non-physiological doses of the natural derivatives.

## 3. Bioaccessibility and Bioavailability of Carotenoids

Overall, the experience acquired to date demonstrates the nutritional relevance of carotenoids, thus imposing the need to take them daily in adequate quantities. However, the functional benefit resulting from carotenoid intake depends not only on the total amount ingested, but also on their bioaccessibility and bioavailability following ingestion, which in turn are mediated by several factors [29,30]. Both Castenmiller and West [31] and van Het Hof and co-workers [32] suggested grouping these factors under the mnemonic SLAMENGHI, an abbreviation that includes species of carotenoids, molecular linkage, amount of carotenoids consumed, matrix, effectors of absorption and bioconversion, nutrient status of the host, genetic factors, host-related factors, and mathematical interactions.

**Figure 2:**
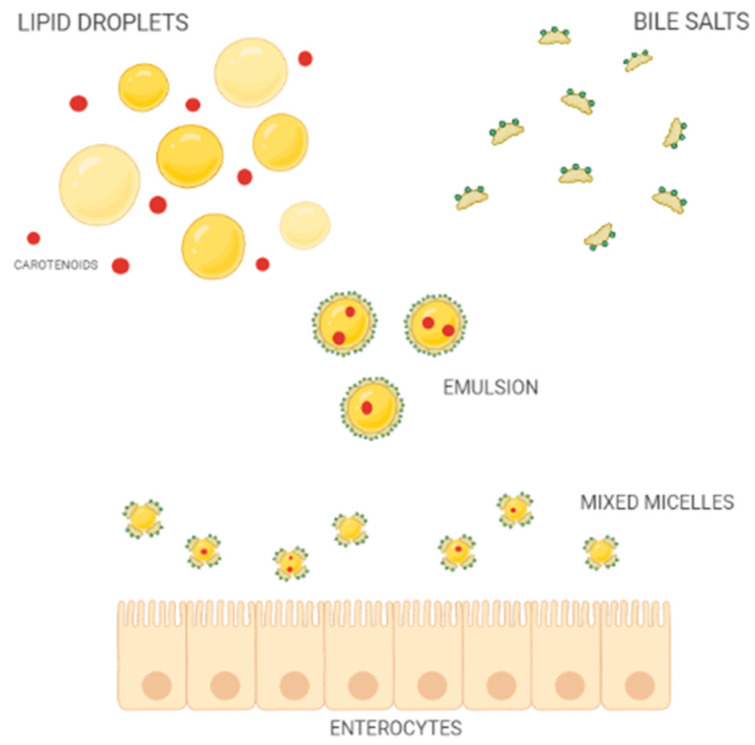
Schematic representation of mixed-micelle formation in the small intestine.

Once released from the food matrix, throughout the action of digestive enzymes of the oral, gastric, and duodenal tracts, carotenoids pour into the intestinal lumen resulting in mixed micelles (Figure 2) together with other lipophilic compounds of the meal, such as cholesterol, fatty acids, acylglycerols, and phospholipids, as well as bile salts of hepatic secretion [33].

The bioaccessibility of carotenoids is defined by the percentage of carotenoids solubilized into mixed micelles [34]. Both the composition and size of micelles affect the absorption process, which in turn impacts the bioavailability of carotenoids [34,35]. Therefore, carotenoid availability depends on several different variables that, on average, do not allow for the absorption of more than 5–30% of the ingested amount [36].

To overcome these limits, different strategies have been pursued over time, the most promising being the incorporation of carotenoids into suitable nanostructured delivery systems. This allows carotenoids to be more easily dispersed into food products, increasing their solubility and, accordingly, their intestinal accessibility and availability [37,38]. Moreover, the nanostructured delivery systems also preserve carotenoids from chemical degradation, ideally maintaining their nutritional value even after the processing, storage, and digestion of the food matrices.

This review summarized the main results achieved in the field of nanostructured systems developed to enclose and deliver carotenoids, improving their bioaccessibility and bioavailability.

Before dealing in detail with bioaccessibility and bioavailability of carotenoids, a distinction between the two concepts must be made. Bioaccessibility can be defined as the solubilized portion compared to the whole amount of nutrient ingested; therefore, the ratio of nutrient available for absorption [39,40]. In the case of carotenoids, and of lipophilic molecules in general, bioaccessibility reflects the ease with which they exit the food matrix and are incorporated into mixed micelles [41]. Therefore, the bioaccessibility of lipophilic molecules (B*) can be defined through the following Equation (1):

B* = 100 × mM/mT(1)

where mM defines the amount of substance included in the mixed micelles and mT represents the total original amount of substance in intestinal fluids [38].

On the other hand, bioavailability can be defined as the portion of the ingested nutrient, in this case of carotenoids, which is available to be used by the human body for normal physiological purposes or for storage [41]. Bioavailability is the result of numerous factors, summarized in the following Equation (2):

BA = B* × A* × D* × M* × E*(2)

where B* represents the bioaccessibility of the molecule, A* is the absorbed fraction (defined by the uptake of the substance by the enterocytes), D* defines the distribution of the substance to the various tissues of the body, M* represents the metabolism of the substance (defined by the activity of chemical reagents or metabolic enzymes present in the human body), and E* represents the excretion of the substance (removal of bioactives and their metabolites from the body through feces or urine).

As for A*, multiple factors must be taken into account. In the past, carotenoid uptake was considered to simply occur by passive diffusion into enterocytes. At present, it is clear that different lipid membrane transporters take part in their absorption, including scavenger receptor class B type 1 (SCARB1), lipid sensor CD36, and NPC1-like transporter 1 (NPC1L1). Moreover, the amount of the absorbed carotenoids is modulated by a fine regulatory network, exerting negative feedback on the activity of membrane SCARB1 by means of the intestine-specific homeobox transcription factor ISX [42,43].

In general, bioaccessibility and absorption occur in the gastrointestinal tract, while metabolism might occur both in the intestine and different sites of the body, and distribution and excretion occur exclusively once the substance has overcome the intestinal barrier. Each of the mentioned factors is time-dependent, which implies a change in the concentration of the substance at the site of action over time [38].

Clearly, bioaccessibility and bioavailability are two strictly connected concepts, as the amount of carotenoids that is utilized by the body depends on their previous release from the food matrix and their absorption in the gut [41]. Additionally, they both influence carotenoid bioconversion, which is the ratio of bioavailable pro-vitamin-A carotenoids converted into retinol [31].

## 4. Factors Affecting the Bioaccessibility and Bioavailability of Carotenoids

### 4.1. Molecular Structure of Carotenoids

The molecular features of carotenoids were observed to influence their bioaccessibility. In particular, significant diversities were detected between carotenes and xanthophylls.

Carotenes, in plant foods, occur especially in the form of large and tightly packed crystalline aggregates; therefore, they are considered to have very low bioaccessibility if not previously heated or consumed together with fats or oils. These crystalline aggregates, in fact, must be disintegrated and dissolved in order to be included in mixed micelles, which represents a further obstacle to overcome in comparison to carotenoids that occur in a pre-dissolved state. Additionally, due to their highly lipophilic nature, in the intestine tract, carotenes are located in the core of lipid droplets, formed following the interaction with bile salts, which makes it more difficult for them to be transferred into the small internal core of mixed micelles.

On the other hand, xanthophylls tend to be less hydrophobic and therefore more bioaccessible than the previously described counterparts. The main reason behind this difference stands in the presence of one or more oxygen atoms in the xanthophyll structure. The most common substitute groups that can be found are hydroxy, methoxy, carbonyl, and epoxy groups. These oxygenated groups make the carotenoid molecule more polar, therefore enhancing the overall solubilization of xanthophylls in the digestive tract facilitating their integration into mixed micelles. Xanthophylls, indeed, are located on the surface of lipid droplets, which allows them to transfer more efficiently into the mixed micelles compared to carotenes [44].

Another structural factor that influences the molecule’s polarity and form is the configuration in which carotenoids occur. In plant food, they are typically found in the all-*trans* isomer form, less bioaccessible and bioavailable compared to the *cis* configuration. The different bioaccessibility and bioavailability are due to the fact that the all-*trans* isomeric form is more prone to crystallization and aggregation than the *cis* counterpart, as the latter tends to be shorter in length and more easily solubilized into mixed micelles. The *cis* isomeric form was observed to augment subsequent to heating, in particular, the incidence of isomerization was observed to be related to the intensity and time exposure to heat treatment [31,45].

In a study led by Schweiggert and co-workers [46], the bioaccessibility and bioavailability of β-carotene and lycopene in carrot, tomato, and papaya were compared. The results obtained show that the bioavailability of β-carotene from papayas is nearly three times greater than that from carrots and tomatoes, while the difference of β-carotene bioavailability between the carrots and tomatoes is irrelevant. An explanation was found in the morphology of chromoplasts and in the physical deposition form of carotenoids. In particular, in papayas, β-carotene is stored in a liquid-crystalline form, while lycopene is deposited as very small crystals; these forms are associated with major bioaccessibility.

By an in vivo study, Van Het Hof and co-workers [32] compared the bioavailability of β-carotene and lutein in a controlled diet. The response from the plasma analysis reported the very low relative bioavailability of β-carotene from mixed vegetables (14%) and a higher bioavailability of lutein (67%), confirming the high diversity in the uptake of the different species of carotenoids, especially between carotenes and xanthophylls.

### 4.2. Esterified Carotenoids

In most fruits and some vegetables, carotenoids that include hydroxy groups in their structure can be found either as free xanthophylls or as carotenoid esters. In fact, a single xanthophyll can be esterified with different fatty acids, forming a wide variety of structures. Furthermore, in carotenoids containing only one hydroxy group, such as β-cryptoxanthin and zeinoxanthin, there is just one position available for acylation with fatty acids, which leads to the exclusive formation of monoesters. On the other hand, molecules, such as zeaxanthin and lutein, hich have two hydroxy groups, can either form monoesters or diesters. Regarding carotenoid diesters, acylated fatty acid can either be the same in both hydroxy groups (homodiester), or they can be different (heterodiester) [47]. On the contrary, carotenes are not able to form esters since they have only carbon and hydrogen in their structure. The fatty acids bound to xanthophylls in fruits are generally saturated, such as lauric, myristic, palmitic, and stearic acids [48].

Concurrently with the ripening of fruit and the senescing of certain vegetables, chloroplasts are converted into chromoplasts and an increment in xanthophyll esterification occurs, while the level of free xanthophylls decreases; therefore, esterification seems to be correlated with the ripening process [49]. This phenomenon does not occur in leafy-green and in dark-green vegetables, where carotenoids are located in chloroplasts and no xanthophyll esters are formed. Regarding the bioavailability of xanthophyll esters, these were observed to be equally or even more bioavailable than the corresponding free carotenoids [47].

### 4.3. Amount of Carotenoids Consumed

It is assumed that the concentration of carotenoids found in human serum is linked with the consumption of foods rich in carotenoids [50]. Several studies showed a variable intake of carotenoid-rich foods throughout different European countries, which resulted in different levels of carotenoids in the bloodstream. In particular, higher concentrations of total carotenoids were observed in the southern European population, rather than in the northern European population [51,52]. Although, in northern countries, the level of carotenoids in vegetarians’ blood was observed to be comparable with that found in southern areas, supporting the finding that vegetarians tend to consume more fruits and vegetables and have higher concentrations of blood carotenoids in comparison to non-vegetarians [52,53]. Furthermore, a less efficient absorption rate was observed at higher doses of β-carotene; for example, in a study by Prince and Frisoli [54], the administration of β-carotene divided into three daily doses corresponding with meals increased serum β-carotene three times more than the same dose administered only once a day.

### 4.4. Food Matrix and Carotenoid Location

Food matrix components and characteristics considerably affect carotenoids’ bioaccessibility and bioavailability. Significant differences in the release of the same carotenoid species were observed between distinct food matrixes.

In green-leafy vegetables, carotenoids are located within chloroplasts, which are subcellular organelles that take part in the photosynthetic process. Due to their hydrophobic nature, they are associated with thylakoids, in which photosystems I and II are located, resulting in large protein complexes. The orientation within the membrane is associated with the polarity of the specific carotenoid and with the nature of the substitutes. As a consequence of this complex and rigid structure, carotenoids that are found in leafy-green vegetables tend to be difficult to extract from the food matrix, causing poor bioaccessibility [44].

On the other hand, in non-photosynthetic plant tissues, carotenoids are located in chromoplasts [44], where they can be found in different physical forms. For instance, α- and β-carotenes exist in a crystalline form in carrots, and the same is also true for lycopene in tomatoes. On the contrary, the latter is dissolved into lipid droplets in orange and yellow fruits (mango, papaya, pumpkins, etc.). The second form is considered the most bioaccessible between the two; yet, in both cases, they are more bioaccessible than when complexed to proteins in chloroplasts, as in leafy-green vegetables [55].

In an in vitro study, Palmero and co-workers [56] analyzed the effect of natural structural barriers, such as cell walls and chromoplasts, on carotenoid bioaccessibility. The used technique consisted of isolating different sections from carrots and tomatoes, characterized by diverse levels of bio-encapsulation (carotenoid-enriched oil, chromoplasts, small cell clusters, and large cell clusters) to compare the bioaccessibility of the target compounds. A considerable reduction in bioaccessibility was observed with the increase in the bio-encapsulation level. Additionally, differences in cell wall material and chromoplast structure were observed to have a determining role. In fact, in carrots, both cell walls and chromoplasts represented a critical obstacle for the release of carotenoids, while in tomatoes, the chromoplast structure was observed to be the main barrier. To this extent, it is important to mention the difference between carrot and tomatoes cell wall materials, observed by Jeffery and co-workers [57], which was observed to be related to the difference in the compounds’ bioaccessibility. In fact, carrot cell walls were very fibrous, compact, and appeared to consist of layers; also, the presence of pectin may influence its porosity by reducing it. On the contrary, cell walls in tomatoes were thinner and less fibrous, while the porosity was higher because of their predisposition to lose cellular adhesion.

Furthermore, even some animal products can be considered as a source of carotenoids, for example, egg yolk, which is a good source of lutein and zeaxanthin. In the case of egg yolk, lutein and zeaxanthin are mostly found within the core of low-density lipoproteins and stored within the membrane. Additionally, relevant amounts of β-carotene were found in dairy products (milk, butter, and cheese), in which they exist in a lipid-dissolved form, which is a highly bioaccessible state [44].

### 4.5. Food Heating and Processing

As previously suggested, in raw plant foods, the rigidity of cell membranes and plant cell walls affects, to a great extent, the possibility of carotenoids to be released from the food matrix and prevents, in part, the action of digestive enzymes. In this regard, food heating and processing were observed to be effective by softening and disrupting cell membranes and plant cell walls, thus allowing a considerable release of carotenoids with a consequent increase in the solubilized fraction. Furthermore, a reduced particle size, induced by mechanical and chemical disruptions, considerably contributed to the improvement of the carotenoids’ bioaccessibility by increasing the surface area accessible to enzymes [45]. Several studies were conducted in order to assess the impact that food heating and processing had on carotenoid bioaccessibility. As an example, Castenmiller and co-workers [55] compared the effects that variously processed spinach products had on serum carotenoid concentrations. They observed that after the consumption of liquefied spinach, in which the food matrix was completely disrupted, the concentration of β-carotene found in the serum was the highest (9.5%) among the ones observed after the consumption of minced (6.4%) or whole-leaf spinach (5.1%). Another study, conducted by Hedrén and co-workers [58], developed an in vitro digestion model to measure the impact of both heating and particle size on the bioaccessibility of α- and β-carotenes in carrots. An increment in the bioaccessibility of β-carotene from 3% (raw, chopped carrots) to 21% was observed after homogenization and to 27% after cooking the pulp. Similar results were also observed for α-carotene. A study by Stinco and co-workers [59] found that industrially processed and hand-squeezed orange juice also differed in bioaccessibility and particle size. Industrial extraction, in fact, produces a smaller particle size than hand-squeezed juice, resulting in greater bioaccessibility; it must be taken into consideration that industrial processing also involves pasteurization, which tends to slightly reduce the bioaccessibility of carotenoids. Therefore, the bioaccessibility of bioactive carotenoids in orange juice appeared to be more associated with mechanical processing than thermal treatment. A review by Palermo and co-workers [60] collected evidence on the effect that cooking exerts on the content of carotenoids (together with other phytochemicals) in vegetables. The two major consequences of cooking phytochemicals in general were observed to be (1) a reduction in the concentration due to thermal degradation and (2) an increment in the extractability due to the softening of the vegetal matrix. Regarding carotenoids, crescent boiling, frying, and microwaving time were associated with a generally lower carotenoid concentration due to degradation. On the other hand, cooking can lead to isomerization, and thus in the transformation of all *trans*- to *cis*-form carotenoids, characterized, as previously explained, by greater bioaccessibility and bioavailability. However, it is important to also consider the fact that both the breakdown of cellulose structures and denaturation of carotenoid–protein complexes induced by heating cause a major and more efficient release of carotenoids from the food matrix.

### 4.6. Dietary Fiber Intake

Apart from the uncountable benefits linked to its adequate intake, dietary fiber may impact nutrient uptake as its presence is associated with a reduction in the rate and, in some cases, the extent of nutrient uptake. Therefore, several effects exerted by dietary fiber tend to reduce carotenoid absorption in the gut, in particular, by physically trapping carotenoids within its structure and by causing an enhancement of the viscosity of gastric fluids, which results in reduced peristaltic mixing, preventing an adequate distribution of the digestive enzymes and bile salts. Furthermore, dietary fiber was also observed to directly bind bile salts, inhibiting diffusion across the unstirred layer [30]. From a study by Riedl and co-workers [61], it emerged that the concentration of carotenoids in plasma varies whether water-soluble dietary fibers (pectin, guar, and alginate) or water-insoluble dietary fibers are consumed; in fact, water-soluble dietary fibers were observed to considerably reduce the relative absorption of β-carotene, while water-insoluble fibers still reduced its absorption, but to a minor extent; the same result was obtained for canthaxanthin. On the other hand, lycopene and lutein absorption was observed to be diminished by both types of fibers in the same way.

Furthermore, a study by Verrijssen and co-workers [62] investigated the effect of pectin type and degree of methyl esterification (DM) on in vitro β-carotene bioaccessibility and lipid digestion in emulsions. Citrus pectin (CP) and sugar beet pectin (SBP) were utilized. In the case of CP-based emulsions containing β-carotene-enriched oil, water, and pectin, β-carotene bioaccessibility and lipid digestion (incorporation of free fatty acids and monoacylglycerols in micelles) were increased with pectin at a higher DM (57%), in comparison to emulsions containing pectin with a lower DM (30%). The same response was not observed in SBP-based emulsions, in which neither β-carotene bioaccessibility or lipid digestion were correlated to pectin DM. These results suggest that DM influences the incorporation into the mixed-micelles process for both β-carotene and lipid, but depending on the pectin source, some other properties may influence the results.

### 4.7. Dietary Minerals Intake

Similarly to what happens with other key nutrients, the intake of minerals may also affect bioaccessibility and bioavailability of carotenoids. Moreover, in the intestinal lumen, mineral cations are likely to complex bile salts and precipitate non-esterified fatty acids, thus reducing the availability of the key components allowing carotenoid emulsification and micellarization as a prerequisite for their uptake.

Biehler and co-workers [63] investigated in vitro the effects of selected divalent ions, such as calcium, magnesium, zinc, and iron, on the micellarization and cellular uptake of spinach-derived carotenoids. Exploiting a digestion model coupled to Caco-2 cells, the authors demonstrated that both the steps were significantly inhibited by the presence of minerals in the digestate, in a dose-dependent manner. The worst effect was achieved with iron. Indeed, at a 12.5 mmol/L concentration, it brought about a remarkable reduction in micellarization and uptake, to 22.5 and 5.0% (*p* < 0.001), respectively, compared to the control. On the contrary, magnesium proved to be less impactful: despite still having detrimental effects, it decreased the uptake value to 69.2% (*p* < 0.001) when used at 25 mmol/L. Similar outcomes were also achieved by Corte-Real and co-workers [64], who investigated in vitro the bioaccessibility of lutein, neoxanthin, lycopene, and β-carotene in the presence of calcium, magnesium, zinc, and sodium, and correlated the observed behaviors with the physical parameters of the digestate. A progressive increase in the concentrations of the ions steadily decreased the viscosity of the medium, in turn reducing carotenoid bioaccessibility. At the same time, the ion increase enhanced the surface tension of the digestate, which in turn modified the carotenoids’ bioaccessibility according to an inverse proportionality relationship. Additionally, the effects of ions proved to be related to the concomitant quantity of bile salts and pancreatic enzymes in the digestate [65], which proved to dampen the negative effects of the added ions when present in relevant concentrations. In a subsequent work [66], the same authors corroborated the negative impact of the same divalent ions on carotenoids contained in large-scale food matrices, such as tomato juice, carrot juice, apricot nectar, spinach, and field salad. Significantly, physiological amounts of calcium and magnesium were sufficient to reduce carotenoid bioavailability, while supplemental doses of zinc were necessary to achieve the same outcomes. On the contrary, the opposite effects were obtained with sodium, which proved to significantly increase the carotenoids’ bioaccessibility when added to most of the investigated matrices.

However, when the authors moved to in vivo studies, enrolling healthy men aged 20–46 years for a randomized, crossover, double-blinded study, they failed to validate the results obtained from the in vitro experiments, as the supplementation of calcium carbonate to spinach-based meals, provided to participants according to a strictly designed test protocol, proved to not modify the availability of carotenoids. Furthermore, comparable amounts of lutein, β-carotene, and β-cryptoxanthin were recovered from the blood samples of all the participants, regardless of whether or not their meals were supplemented with calcium [67].

Clearly, the experimental evidence collected to date is not sufficient to understand the role of dietary minerals in carotenoid bioaccessibility and bioavailability, also considering the complexity of composition of the intestinal lumen and mutual interactions between the single components. Further investigations are necessary, and those performed in vivo are highly desirable, being the ones allowing us to achieve a clear picture of the dynamic processes occurring in the gut.

### 4.8. Dietary Fat Intake

Since carotenoids are hydrophobic molecules, their uptake not only relies on their release from the food matrix, as previously stated, but also the efficiency of the solubilization process, by bile acids and digestive enzymes, and the consequent micellization. To this extent, dietary lipids are considered as important adjuvants for carotenoid bioaccessibility and bioavailability, mostly in carotenoid-rich fruits that contain a very low fraction of lipids [68]. Therefore, the presence or addition of oils and fats can increase carotenoid bioaccessibility by enhancing the dispersion of carotenoids, thus promoting their solubilization and emulsification in the digestive tract [39]. Furthermore, the addition of dietary lipids also enhances the secretion of bile salts and triglyceride-cleaving lipases, therefore causing an increment in the presence of emulsifiers that promote the micellization process [44]. A minimum amount of fat is therefore fundamental for the uptake of carotenoids; an optimal absorption rate was observed to occur with an intake of fat of at least 5 g per day [55].

In order to maximize the uptake of carotenoids, fat and carotenoid sources should be provided in the same meal; otherwise, it is still possible that due to delayed gastric emptying or delayed carotenoid absorption, fat ingested from a later meal may still enhance the inclusion of carotenoids into mixed micelles. Additionally, fat obtained from a previous meal may persist in the intestine and boost carotenoid absorption, even if the latter was ingested hours later [69]. However, the time that elapses between the ingestion of the carotenoid and fat sources cannot be too extended; in fact, it was observed that subjects who consumed fat sources 16 h after the intake of a β-carotene dose did not show an increment in serum β-carotene [70].

A study conducted by Nagao and co-workers [39] showed an improvement in the bioaccessibility of β-carotene in spinach following the addition of various fats, oils, and long-chain triacylglycerols; the same result was not observed with lutein, which is less hydrophobic than β-carotene. Similarly, free fatty acids, monoacylglycerols, and diacylglycerols also improved the bioaccessibility of β-carotene.

Furthermore, Liu and co-workers [71], using a gastrointestinal model, evaluated the different effect that oil type has on the bioaccessibility of carotenoids from yellow peppers. Medium-chain triglycerides (MCTs), long-chain triglycerides (LCTs), and indigestible orange oil (OO) were considered. Carotenoid bioaccessibility was proven to be impacted by oil type in the following order: LCT > MCT > OO > control (no oil). In particular, a significant increment in the bioaccessibility of carotenoids was observed in the presence of LCT and MCT; this result is ascribable to the presence of the free fatty acids produced from lipid digestion, which were included in the micelles with bile salts, improving the solubilization ability of the intestinal fluids; on the contrary, the digestion of OO did not produce any free fatty acids. Therefore, in this case, carotenoids were included in mixed micelles that only contained bile salts. Furthermore, the greater influence that LCTs had on carotenoid bioaccessibility, compared to that of MCTs, can be explained by the greater solubilizing capacity of micelles formed by long fatty acids.

Kopec and co-workers [72], in a in vivo study on human subjects, detected an increase in the absorption of provitamin-A carotenoids obtained from tomato sauce and raw carrots, when combined with avocado. Therefore, the presence of a lipid-rich food was observed to maximize the provitamin-A uptake in the intestine and enhanced the conversion of carotenoids into vitamin A. The concentrations of carotenoids and vitamin A were measured in the TRL fraction.

In an in vitro study conducted by González-Casado and co-workers [73], the addition of different oils to the carotenoids from tomato derivates (puree and tomato cubes) was correlated with their bioaccessibility. Total carotenoid and lycopene bioaccessibility were considered. The addition of 5% of oil caused an increase in both total carotenoid bioaccessibility and lycopene bioaccessibility; in particular, in tomato samples without oil, the release of carotenoids from the matrix ranged from undetectable values to 2.9 ± 0.4% for total carotenoids and 1.8 ± 0.2% for lycopene. Following the addition of different types of oils, peaks of 29.3% for total carotenoids and 27.2% for lycopene were obtained. These maximum values corresponded to the utilization of tomatoes in puree form and the addition of olive oil. Therefore, in both cases, the greatest increase in bioaccessibility was observed following the addition of olive oil, followed by sunflower and coconut oils. The diversities between the different types of oil may be associated with both the chain length of fatty acids and their degree of unsaturation; oils rich in long-chain fatty acids (such as olive and sunflower oils) were observed to enhance more carotenoid inclusion in mixed micelles compared to oils rich in medium-chain fatty acids (such as coconut oil). Similarly, oils rich in unsaturated fatty acids (such as olive and sunflower oils) were demonstrated to be more efficient in enhancing carotenoid micellization if compared to oils rich in saturated fat (such as coconut oil).

### 4.9. Dietary Protein Intake

Being characterized by both hydrophobic and hydrophilic portions, proteins are claimed to be amphiphilic compounds. Therefore, they have emulsifying properties that turn out to be crucial for foodstuffs. Indeed, these macronutrients play a key role not only in the setting up of food matrices, but also in the digestion and absorption of their constituents. This is especially important for the lipophilic carotenoids that, upon dietary intake, must first dissolve in lipid droplets, in the stomach, and then produce mixed micelles in the small intestine, to be absorbed.

As thoroughly reviewed by Iddir and co-workers [74], peptides resulting from protein digestion affect the solubilization and stability of carotenoids as lipid droplets, modifying their assembling into mixed micelles. However, the rebound on the bioaccessibility and bioavailability of the natural compounds cannot be predicted a priori. Indeed, distinctions must be made on the basis of both the type and concentration of peptides, as they emerge from protein digestion, as well as the nature of carotenoids.

Sodium caseinate (SC), whey protein isolate (WPI), soy protein isolate (SPI), and gelatin (Gel) were investigated in vitro for their ability to modify bioaccessibility when added in increasing concentrations to oily solutions of selected, isolated carotenoids, such as β-carotene, lycopene, and lutein [75]. As for peptides, and by referring to β-carotene, the best effect was observed with SC, which proved to significantly increase the bioaccessibility of the natural compound. WPI and Gel turned out to be less powerful, although they still exerted positive effects. On the contrary, the use of SPI resulted in a reduction in bioaccessibility. Regarding carotenoids, and by referring to the most performing peptides, opposite trends were observed when comparing the bioaccessibility of the more lipophilic β-carotene with the more polar lutein. Moreover, while the former markedly increased, the latter turned out to decrease up to 50%. Moreover, the effects of protein isolates were observed to also depend on digestive conditions, as the greatest increase in bioaccessibility was obtained with the better digested proteins. Indeed, a more marked proteolysis affects the macroviscosity, surface tension, and emulsification of the resulting digestate, thus fostering the micellization of carotenoids and, ultimately, their bioaccessibility [76].

Moving from protein isolates to peptide-rich matrices, such as cod and turkey, as well as from single carotenoids to carotenoid-rich matrices, such as tomato juice, carrot juice, and spinach, similar though less marked behaviors were observed. Additionally, in this case, the positive effects of protein matrices on carotenoid bioaccessibility were more pronounced for tomato juice, made of nonpolar carotenes, than for carrot juice and spinach, characterized by more polar compounds, such as xanthophylls [77].

A recent crossover, randomized trial conducted on healthy volunteers demonstrated that, first in this field, WPI and SPI were truly able to impact the post-prandial bioavailability of carotenoids obtained from a tomato–carrot juice mixture. Additionally, in vivo, the better-digested WPI proved to enhance carotenoid bioavailability, while the less hydrolyzed SPI led to negative effects [78].

## 5. Formulation of Nanostructured Delivery Systems

As previously discussed, carotenoids have considerable health benefits. However, their low bioavailability from natural sources (5–30%) prevents a large portion of the ingested nutrient to be actually used. Therefore, in order to overcome this limit, different strategies for the extraction and isolation of carotenoids as well as their encapsulation in various delivery systems were developed [79].

Carotenoids can be incorporated into foods by their inclusion in emulsions or nanostructured delivery systems, which if needed can be further encapsulated by drying processes. These techniques have the advantage to permit carotenoids to be more easily dispersible into food products, gain more stability, and enhance their bioactivity [80]. The creation of food-grade delivery systems requires the utilization of GRAS (generally regarded as safe) ingredients, which must be previously recognized as appropriate for human consumption, for example, by the EFSA (European Food Safety Authority) in Europe or by the FDA (United States Food and Drug Administration) in the USA [81,82]. Furthermore, delivery systems should also be food-matrix compatible, which means they should not alter the original appearance, flavor, or texture of the final product. Finally, it must also be considered that the delivery system, once incorporated into food products, must undergo the same processing operation of the food product itself during its production, storage, distribution, and utilization; therefore, they must be able to resist the environmental changes that can occur during these phases, such as heating, freezing, dehydration, and pH variations [83].

Delivery systems should possess different functional characteristics that must be taken into account during the selection of the most suitable nanocarrier to use [83]. These include:

- Loading capacity (LC): the relation between the amount of encapsulated material and mass of carrier material; ideally, a delivery system should have a high LC; hence, it should be able to encapsulate as much material as possible.
- Loading efficiency (LE): reflects the ability of a delivery system to retain encapsulated molecules over time. During the production, storage, and transport steps, part of the carried material can be released from the delivery systems; therefore, ideally, the loading efficiency should be high.
- Delivery efficiency (DE): assesses the ability of a delivery system to carry the encapsulated compound to a specific site of action. Even in this case, delivery efficiency should be high.
- Delivery mechanism: once at the site of action, the material can be released either gradually or in response to specific environmental triggers.
- Protection against chemical degradation: chemical degradation may occur under different forms, such as oxidation, hydrolyzation, and isomerization, which can eventually lead to a loss in bioactivity. Chemical degradation can be induced and accelerated by factors, such as heat, light, oxygen, and pH variations, and can be managed through the encapsulation of the compound of interest.
- Bioaccessibility/bioavailability: delivery systems should enhance the encapsulated compound’s bioaccessibility and bioavailability.

To encapsulate carotenoids, several delivery systems can be used, which can be broadly divided into two categories, lipid-based and biopolymeric nanocarriers, thoroughly reviewed hereafter.

## 6. Lipid-Based Nanocarriers

Lipid-based delivery systems aim to enhance the solubilization and micellization of the carried carotenoids when compared to the crystalline form in which they occur in vegetable tissues. Additionally, the presence of lipids in the formulation promotes the further secretion of bile and pancreatic juices, thus supporting lipid digestion and carotenoid absorption. The bioaccessibility of carotenoids can be managed through the selection of proper formulation ingredients and the final structure of the nanocarrier. Furthermore, it is critical to create systems with the smallest dimensions as possible and that can resist gastrointestinal (GI) conditions (low pH and GI enzymes) [84]. The most promising lipid-based nanocarriers for carotenoid delivery developed to date are described in detail below (Table 1).

### 6.1. Nanoemulsions and Microemulsions

**Figure 3:**
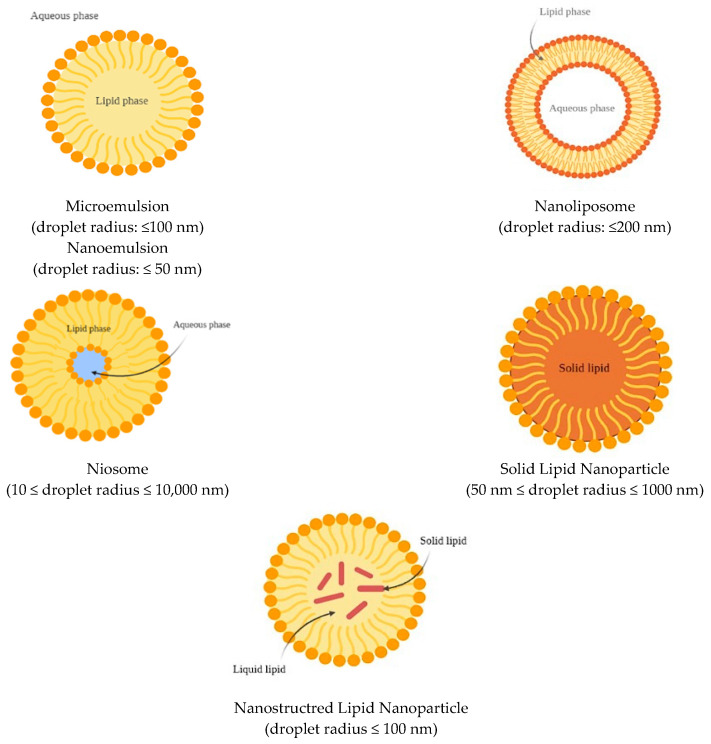
Schematic representation of lipid-based nanocarriers.

Nanoemulsions and microemulsions (Figure 3) are widely used as vehicles to deliver lipophilic bioactive molecules into mainly water-based beverages and industrial foods [85]. They consist of a combination of two immiscible liquid phases, namely, oil (O) and water (W), where one liquid is dispersed as spherical or spheroidal droplets into the other. Emulsions can either be water in oil (W/O) or oil in water (O/W), and the combination of multiple phases can also produce double emulsions, such as W/O/W and O/W/O [86].

Nanoemulsions are defined as emulsions characterized by a droplet radius ≤ 50 nm, while microemulsions have a droplet radius ≤ 100 nm. Because of the small size of the droplets in comparison to the wavelengths of light (r << λ), they can appear as transparent if the radius is < 30 nm, or opalescent/opaque if their radius is > 30 nm. Nanoemulsions are thermodynamically unstable and therefore undergo gravitational separation (creaming or sedimentation), flocculation, coalescence, or Ostwald ripening [87,88]. However, their breakdown can be retarded by using selected additives, including emulsifiers (added to stabilize the interfaces), stabilizers (used to modify the viscosity of the continuous phase), and weighing agents for the oil phase, which reduce the density difference between continuous and dispersed phases [89]. In contrast, microemulsions are thermodynamically stable at a constant pressure and temperature.

Nanoemulsions and microemulsions can be obtained by utilizing either high-energy approaches based on disruptive mechanical forces, such as homogenization, microfluidization, and sonication, or low-energy approaches, such as spontaneous emulsions (SEs), emulsion phase inversion (EPI), phase inversion temperature (PIT), and phase inversion composition (PIC), which rely on the physicochemical or molecular geometry changes of the emulsifier induced by temperature, ionic force, or relative ratio among the ingredients [90]. The appropriate method is chosen by considering the properties of the surfactant and oil phase and the desired final characteristics of the obtained emulsion. In general, low-energy methods are more likely to create small droplet sizes when compared to high-energy methods. However, low-energy methods have some limitations, such as the high concentration of emulsifier (which can influence the taste, safety, and costs) and the type of oils and emulsifiers that can be added. For instance, protein and polysaccharide emulsifiers cannot be used to produce nano- and microemulsions through low-energy methods. On the other hand, high-energy approaches are more versatile and compatible with the use of various oils and emulsifiers [87,91].

In food matrices, nanoemulsions and microemulsions are primarily used for the encapsulation and delivery of lipophilic compounds, such as carotenoids. Therefore, they consist of O/W systems. Due to the small particle size and high surface-to-volume ratio, these systems can improve carotenoid bioavailability while preserving their chemical stability [89,92].

The lipophilic compound of interest is generally included in the oil phase before emulsification. Once the emulsion is formed, its location within the droplet is determined by its molecular and physiochemical characteristics. Less polar molecules tend to locate in the inner core of the droplet, while more polar molecules can also locate within the amphiphilic shell [87].

The choice of the carrier oil can affect emulsion properties. For example, a study conducted by Zhou and co-workers [93] compared the influence that different carrier oils (in particular palm, coconut, fish, and corn oils) have on the stability and in vitro digestibility of β-carotene-based nanoemulsions. The bioaccessibility of the compound was greater if palm and corn oils were used, while bioaccessibility decreased with fish and coconut oils. Furthermore, the particle diameter was also affected by the different carrier oils; emulsions created with palm oil formed the smallest droplets (168 nm), followed by coconut oil (173 nm), corn oil (177 nm), and fish oil (185 nm). This was also an important indicator, as bioaccessibility was observed to increase with smaller particle sizes. The different emulsions were also monitored through a lapse of 42 days of storage; β-carotene in unsaturated oils, such as fish and corn oils, was more prone to degradation caused by oxidation. Overall, palm oil was the most suitable to be included in β-carotene emulsions.

A similar study conducted by Yi and co-workers [94] on six different oil types (corn, olive, canola, palm, coconut, and medium-chain triglycerides (MCTs)) described nanoemulsions with a diameter smaller than 200 nm and incorporating β-carotene. The amount of β-carotene included in the mixed micelles was positively correlated with the length of the fatty acids composing the oil. Moreover, oils rich in unsaturated fatty acids enhanced β-carotene micellization.

Compared to carotenes, there is much less evidence for the encapsulation of xanthophylls in nanoemulsions [85]. A study by Liu and co-workers [95] evaluated astaxanthin bioaccessibility when included in nanoemulsion delivery systems containing long-chain triglyceride (LCT) oils, such as olive, flaxseed, and corn. As in the previous studies, the bioaccessibility of astaxanthin was influenced by the oil type and was dependent on the unsaturation and chain length.

The selection of a suitable emulsifier is also important when delivering carotenoids though nanoemulsions. A recent study [96] compared the use of four different types of the following protein emulsifiers: peanut protein isolate (PPI), soy protein isolate (SPI), rice bran protein isolate (RBPI), and whey protein isolate (WPI). All the nanoemulsions achieved high β-carotene-encapsulation levels. The PPI-emulsified nanoemulsion displayed the smallest droplet size, the greatest stability during storage, and the highest lipolysis rates and bioaccessibility. The smaller particle size of the nanoemulsion probably permitted a greater exposed surface for digestive enzymes.

Surh and co-workers [97] used different surfactants (Tween 20, 40, 60, 80, and 85) and carrier oils to obtain lutein-loaded nanoemulsions. Tween 80 yielded the most stable and bioavailable product. Hou and co-workers [98] also investigated the impact of emulsifier on the release efficiency of β-carotene. In this case, WPI, soybean soluble polysaccharides, and decaglycerolmonolaurate were utilized to formulate β-carotene emulsions. Through an in vitro digestion model, they determined that the emulsifier type had a considerable impact on the release process, and observed that the micellization rates of β-carotene in emulsions stabilized with whey protein isolate, decaglycerolmonolaurate, and soybean soluble polysaccharides were 34.0%, 24.1%, and 21.8%, respectively. Zhang and co-workers [99] created β-carotene-loaded microemulsions utilizing anhydrous milk fat (AMF) and Tween 80. The compound was encapsulated using the phase-inversion temperature method and was notably more stable in AMF-based microemulsions than in soybean-oil-based microemulsion.

### 6.2. Nanoliposomes

Liposomes (Figure 3) consist of concentric phospholipid bilayered vesicles and are characterized by an aqueous core. These structures can entrap and carry both lipophilic and hydrophilic molecules [100]. Lipophilic molecules can be located between the two phospholipid layers, while the aqueous core can host hydrophilic molecules [101]. Liposomes can be distinguished as uni- or multilamellar based on the number of lipid bilayers that are present in the system [86].

The main surfactants used to produce liposomes are phospholipids. They are isolated from lecithin derived from different seed oils, such as soy, sunflower, and canola oil, or from egg yolks and milk. Usually, hydrogenated and saturated phospholipids have a stiffer conformation, which favors liposome absorption in the gastrointestinal tract. In contrast, unsaturated phospholipids make more flexible membranes that are ideal for intraoral absorption. Furthermore, co-surfactants can be used to improve the stability of nanoliposomes; the most commonly used co-surfactants in food matrices are PEGs, MCTs, and cholesterol [101]. Additionally, repulsive electrostatic charges can be created on the surface of the vesicles through the utilization of cationic or anionic phospholipids to inhibit the aggregation, fusion, and sedimentation of nanoliposomes [102]. Lastly, the outer surface can also be covered to improve muco-adhesion using alginates, chitosan, and hyaluronic acid [101].

Liposomes and nanoliposomes are characterized by the same physical, structural, and thermodynamic properties, which are mostly defined by their components and suspension media. However, in nanoliposomes, smaller dimensions provide an increased surface-to-volume ratio, potentially improving the bioaccessibility, bioavailability, and stability of the encapsulated compounds [102]. Furthermore, nanoliposomes require more energy for creation because of the resulting smaller sizes. There are different production methods that can be adopted and are divided into non-mechanical (such as the injection method, diminution of assorted detergent-lipid micelles, freeze drying–rehydration, freeze–thawing, and reverse-phase evaporation) and mechanical approaches (such as microfluidization, colloid mills, high-pressure homogenization, extrusion, and sonication) [86].

The selection of the most appropriate method must consider the characteristics of the encapsulated molecule and its route of application, the physiochemical properties of the chosen suspension medium, the desired characteristics of the final product, and the potential toxicity and actual concentration of the materials that are included in the vesicles [102].

Nanoliposomes are suitable for the encapsulation and delivery of carotenoids. Tan and co-workers [103] successfully encapsulated carotenoids (lutein, β-carotene, lycopene, and canthaxanthin) in liposomal structures, and investigated in vitro the relationships between carotenoid structure and concentration inside the vesicles and bioaccessibility. The greatest bioaccessibility was observed for lutein, followed by β-carotene, lycopene, and canthaxanthin. The bioaccessibility was also observed to be connected to the inclusion ability of the single carotenoid into the lipid bilayer and the concentration of the molecule inside the vehicle and the nature of the delivery system. Carotenoid bioaccessibility and micellization content were lower when the amount of compound in the delivery systems was higher. The same authors [104] also observed that the encapsulation of carotenoids (lutein, β-carotene, lycopene, and canthaxanthin) in liposomes enhanced their antioxidant activity, following the order of lutein > β-carotene > lycopene > canthaxanthin. Furthermore, lutein and β-carotene not only prevented lipid peroxidation, but also protected lipids from pro-oxidants. Instead, lycopene and canthaxanthin showed low protection levels against lipid peroxidation and, to some extent, presented a pro-oxidant effect. These results can be interpreted through a correlation with the incorporation efficiency of these carotenoids into the phospholipid membrane and their effects on membrane dynamics and structure.

Hamadou and co-workers [105] successfully created β-carotene-loaded nanoliposomes using egg and marine phospholipids as compositional ingredients. Marine phospholipids were more efficient for encapsulation than the counterparts. Marine phospholipids also yielded nanoliposomes with a lower mean size and polydispersity index, better capacity in inhibiting lipid peroxidation, and better stability at 4 °C over 70 days.

### 6.3. Niosomes

Niosomes (Figure 3) are bilayered vesicles composed of non-ionic surfactants. Their structure resembles that of liposomes and can therefore encapsulate both lipophilic and hydrophilic molecules [86], and either be uni- or multilamellar [106]. However, the ingredients used in the niosome formulation confer greater physicochemical stability in comparison to liposomes. The main difference relies on the type of surfactant used in the formulation. Liposomes require the use of neutral and ionic phospholipids, while non-ionic surfactants are used for niosomes, which show good biocompatibility and low toxicity [107]. Examples of non-ionic surfactants include derivates of alkyl ethers (such as Brij), alkyl esters (such as Span), and polyoxyethylene sorbitan fatty acid esters (such as Tween). Additives can also be included in the membrane of niosomes, such as cholesterol, which interacts with surfactants and enhances the stability of the system. As in liposomes, charging agents can be added to stabilize the system to prevent aggregation. Furthermore, niosomes are fabricated through simple methods and are less expensive to produce than liposomes [108]. Niosomes can be obtained using thin-film hydration, dehydration–rehydration, hand shaking, ultrasonication, reverse-phase evaporation, “bubble”, ether injection, microfluidization, heating, and freeze–thawing techniques [84].

Niosomes can be classified as small unilamellar vesicles (SUVs, diameters between 10 and 100 nm), large unilamellar vesicles (LUVs, diameters between 100 and 300 nm), and multi-lamellar vesicles (MLVs) that have more than one bilayer [86]. They were originally introduced in the cosmetic industry then adopted in drug delivery as an alternative to liposomes due to the numerous advantages described above.

Regarding the use of niosome for carotenoid delivery, Pallozza and co-workers [109] developed β-carotene-loaded niosomes able to solubilize and deliver the bioactive molecule to cultured cells. They were obtained using Span 40, 60, and 80, and Tween 20, 40, and 60, and cholesterol through the thin-film hydration method. The resulting systems showed high resistance to sunlight, high temperatures, and induced oxidative stress. Furthermore, β-carotene was stable in culture medium up to 96 h and was effectively taken up by cultured cells at concentrations covering the range of physiological levels (0.1–2 µM).

In 2016, Sharma and co-workers [110] formulated lycopene-loaded niosomes to preserve their activity and enhance their bioavailability. The in vitro anti-proliferative efficacy of the formulation was also tested. Niosomes were characterized through in vitro studies, while bioavailability was assessed in vivo. A novel approach was used to develop these delivery systems, consisting of the absorption of pure lycopene extract in glass wool through the absorption–hydration method with the successive formulation of niosomes. Lycopene was stable and resistant to oxidative stress provided by free radicals. Furthermore, the innovative formulation approach produced uniform-sized particles with a high entrapment efficiency. The in vitro release profile was gradual and prolonged, which are important factors for successful treatment. Bioavailability was enhanced and led to a clear increase in blood plasma levels (297.19%). On the whole, niosomes can be considered as an efficient delivery system for carotenoids and lipophilic antioxidants in general.

### 6.4. Solid Lipid Nanoparticles (SLNs)

SLNs (Figure 3) are spherically shaped colloidal delivery vehicles made of lipids that are solid both in the bodily environment and at room temperature [86]. Therefore, the lipid droplets are completely crystallized, with the encapsulated molecules being part of the lipid matrix. Consequently, the mobility of the compound depends on the physical state of the lipid matrix [111]. This characteristic allows for a more controlled and gradual release of the bioactive compound and a lower and prolonged diffusion rate, which provides a better targeted delivery [112]. On the other hand, crystalline structures reduce the loading capacity of these carriers in comparison to other delivery systems [111]. SLNs can be made of a single lipid species or mixtures of different types of lipids [113], with the most common being triglycerides (TAGs) (trilaurin, trimyristin, tripalmitin, and tristearin). However, fatty acids, steroids, waxes, monoglycerides, and diglycerides are also widely used because of their safety for human consumption [114,115,116]. The lipid content in the emulsion system should not be greater than 5–10%, since excessive content can affect both the final particle size and their size distribution. Furthermore, the lipid melting point has a considerable influence on the final particle size. For SLNs, the melting point of the carrier lipids must be higher than room temperature, even if not too high, since lipids that are characterized by high melting points also usually have high viscosity properties, which is a factor that negatively affects the homogenization process and results in larger particles. Furthermore, the lipid type can also affect additional properties of SLNs, such as the crystallization rate, hydrophobicity, and crystal morphological features [116].

The formulation of SLNs includes the use of surfactants and co-surfactants that stabilize the lipid matrix, influence the size of the nanoparticle, and contribute to the crystallization process. Representative examples of the most used surfactants include cetylpyridinium chloride (CPC), Poloxamer 407, and Tween 80 [112,117]. Additionally, the use of solid instead of liquid lipids increases the stability of the encapsulated bioactive compounds, reducing their degradation rate [117].

These structures can carry both hydrophilic and lipophilic molecules and their particle size ranges between 50 and 1000 nm [84]. SLNs can be diversified according to the site of the particle in which the encapsulated compounds are located. These can be dispersed in the core (drug-enriched core), in the outer shell (drug-enriched shell), or can be evenly distributed in the matrix [116].

The main preparation techniques include temperature-controlled high-pressure homogenization (depending on the melting temperature of the lipid phase), oil–water microemulsion breaking, solvent-emulsification diffusion, solvent injection, W/O/W double emulsion, ultrasonication, supercritical fluid, membrane contractor, electrospray, and preparation of semisolid–solid lipid nanoparticles [112].

An in vitro study conducted by de Abreu-Martins and co-workers [118] described the use of different types of lipids for the formulation of β-carotene-loaded SLNs and investigated their effects on the overall digestibility of the obtained particles and β-carotene bioaccessibility. SLNs were prepared by using blends of medium-chain triglyceride (MCT) oil and two different types of solid lipids (glyceryl stearate (GS) and partially hydrogenated palm oil (HPO)). Liquid lipid nanoparticles (LLNs) were also prepared using pure MCT. SLNs prepared using GS were completely digested, as were LLNs. Instead, SLNs fabricated with HPO had higher β-carotene bioaccessibility, which is associated with the higher amount of monounsaturated fatty acids in the micelle fraction.

Mehrad and co-workers [119] successfully produced β-carotene-loaded SLNs containing palmitic acid and corn oil, stabilizing them with WPI. Palmitic acid in the solid state formed a shell around the β-carotene. The use of WPI was observed to improve the physical stability of SLNs and the oxidative stability of β-carotene.

Salminen and co-workers [120] analyzed the impact of the chemical structure of the encapsulated lipid compounds on the structural disposition of SLNs. Functional lipids, including crystalline vitamin-A acetate and β-carotene, were considered. The results achieved reveal that the major influence on the structural arrangement and chemical stability of the encapsulated molecules can be attributed to the solubility of the functional lipids in the aqueous phases and their crystallization temperature in relation to that of the carrier lipid. Furthermore, vitamin A accumulated more on the surface of the nanoparticle and oxidized faster than β-carotene, which was located in the core of the nanoparticle. This is due to the highly lipophilic nature of β-carotene, in contrast to the less hydrophobic nature of vitamin A. Therefore, the stability of β-carotene was observed to be much more dependent on the polymorphic stability and loading capacity of the carrier lipid, in comparison to vitamin A.

Qian and co-workers [116] evaluated the effect that the physical state of a lipid may have on particle aggregation and β-carotene degradation. Both LLNs and SLNs were produced using a mixture of cocoa butter and hydrogenated palm oil as the lipid phase. LLNs were observed to have better stability to droplet aggregation than SLNs after 8 days of storage, since SLNs exhibited a considerable increase in the particle diameter, possibly caused by a change in the morphology or aggregation of the SLNs during storage. Furthermore, the rate of β-carotene degradation (measured through color loss) was also observed to be higher in SLNs than LLNs. This effect was explained by the expulsion of β-carotene from the nanoparticle as the lipid-phase crystallization occurred, causing greater exposure of the compound to pro-oxidants. Therefore, this study highlighted some relevant limitations associated with the use of SLNs in comparison to LLNs.

### 6.5. Nanostructured Lipid Carriers (NLCs)

Nanostructured lipid carriers (Figure 3) are a modification of SLNs in which the lipid phase is formed both by solid and liquid lipids at room and body temperatures [111]. The goal of NLCs is to address some limitations of SLNs, such as the limited loading capacity, drug expulsion during storage, and less water in the dispersion, and to enhance the controlled release profile of active compounds in gastrointestinal conditions [86,114]. Three different NLCs conformations are possible, including imperfect, amorphous, and multiple types [86]. The imperfect type is formed by structurally different lipids that create an imperfect crystal order of lipid nanoparticles upon mixing. This results in the formation of a disordered pattern, which presents gaps that increase the capacity of bioactive compounds to enter the matrix [120,121]. The amorphous type is constituted by a solid but shapeless structure achieved by the mixture of solid lipids and special lipids (such as hydroxyoctacosanylhydroxystearate, isopropylmyristate, or MCTs), which do not crystallize as SLNs [111,121]. Finally, the multiple type is formed by multiple oils in fat and water (i.e., O/O/W emulsion). The solid matrix contains small, liquid, oily nano-compartments, in which the bioactive compound may be dissolved. The solubility is higher in these compartments and a greater loading capacity is allowed. The oily nano-compartments are contained in a solid matrix that facilitates controlled release and preserves the encapsulated bioactive from decomposition [111,114,121].

Most NLC production techniques are similar to those of SLNs [121]. The hot homogenization method is mostly used for the formulation of both NLCs and SLNs. For example, the melting dispersion method requires the bioactive compound and solid lipid to be melted in an organic solvent and then added to a small volume of water phase heated at the same temperature. Stirring at a high speed yields an emulsion that is cooled to room temperature. Furthermore, a rapid and easy method is solvent injection, where lipids are dissolved in a water-miscible solvent and injected by a needle into a stirring aqueous solution [112,121].

Lacatusu and co-workers [122] used squalene (Sq) and grapeseed oil (GSO) to obtain biocompatible antioxidant NLCs enclosing β-carotene. Glyceryl stearate (GS) and n-hexadecyl palmitate (CP) were used as the solid lipids. The smallest lipid nanoparticles (85 nm for GSO and 89 nm for Sq) were obtained using Tween 20 as the main surfactant. Both the delivery systems were characterized by great physical stability. NLCs containing Sq and GSO enhanced the antioxidant properties of the system, whereas NLCs produced with GSO and Tween 80 had the greatest antioxidant activity towards free oxygen radicals (+35% in comparison to pure β-carotene). Additionally, β-carotene-loaded NLCs revealed antibacterial activity against *Escherichia coli* and also showed a correlation between the concentration of β-carotene and that of the liquid lipid rather than particle size.

Recently, Sirikhet and co-workers [123] encapsulated lycopene from *Citrullus lanatus* (watermelon) extract in NLCs through the hot homogenization technique to enhance its stability. Cocoa butter was used as the solid lipid and grapeseed oil was used as the liquid lipid. Additionally, Span 80 and Planrasens^®^ HE20 were utilized as emulsifiers. The small, spherical NLCs particles obtained entrapped lycopene effectively. NLCs were observed to be efficient in protecting the encapsulated compound and maintained their stability when stored at 4 °C for 3 months. Hence, this NLC formulation was observed to be effective as a delivery system for unstable molecules.

**Table 1:** Summary of different studies testing the encapsulation of carotenoids in lipid-based nanocarriers.

Delivery System	Loaded Compound	Emulsifier and Additives	Lipid Phase	Study Outcomes	Reference
Nanoemulsion	β-carotene	Whey protein isolate	Palm oil/ coconut oil/ fish oil	NPs with palm oil were the smallest and had the highest bioaccessibility. After 42 days of storage, β-carotene was seen to be more prone to degradation in unsaturated oils. Palm oil was the most suitable carrier.	Zhou et al. [93]
Nanoemulsion	β-carotene	Sodium caseinate	Corn oil/ olive oil/ canola oil/ palm oil/ coconut oil/ MCTs	Amount of beta-carotene included is positively proportional to the length of the fatty acids. Oils rich in unsaturated fatty acids enhanced β-carotene micellization.	Yi et al. [94]
Nanoemulsion	Astaxanthin	Sodium caseinate + phosphate buffer	Olive oil/ flaxseed oil/ corn oil	Bioaccessibility depended on the unsaturation and chain length.	Liu et al. [95]
Nanoemulsion	β-carotene	Peanut protein isolate (PPI)/ soy protein isolate (SPI)/ rice bran protein isolate (RBPI)/ whey protein isolate (WPI)	Corn oil	All four NPs achieved high encapsulation levels. PPI-emulsified nanoemulsion had the highest lipolysis rates, bioaccessibility, smallest droplet size, and highest stability during storage.	Liu et al. [96]
Nanoemulsion	Lutein	Tween 20/ Tween 40/ Tween 60/ Tween 80/ Tween 85	MCT oil	Nanoemulsion stabilized with Tween 80 was he most stable and bioavailable.	Surh et al. [97]
Nanoemulsion	β-carotene	Whey protein isolate/ soybean soluble polysaccharides/ decaglyceromonolaurate	MCT oil	The emulsifier had a considerable impact on the release process and micellization rates of β-carotene in emulsions stabilized with whey protein isolate, decaglycerolmonolaurate, and soybean soluble polysaccharides were, respectively, 34.0%, 24.1%, and 21.8%.	Hou et al. [98]
Liposome	Lutein/ β-carotene/ lycopene/ canthaxanthin	Egg yolk phospholipid + Tween 80		Bioaccessibility observed: lutein > β-carotene > lycopene > canthaxanthin. Bioaccessibility was connected to the inclusion ability of the carotenoid into the lipid bilayer, the concentration of the molecule in the vehicle, and the nature of the delivery system.	Tan C. et al. [103]
Liposome	Lutein/ β-carotene/ lycopene/ canthaxanthin			Encapsulation of carotenoids into liposomes enhanced their antioxidant activity. The strongest activity followed the order: lutein > β-carotene > lycopene > canthaxanthin. Lutein and β-carotene also protected lipids from pro-oxidant elements.	Tan C. et al. [104]
Nanoliposome	β-carotene	Marine phospholipids/ egg phospholipids		Marine phospholipids were seen to be more suitable for the creation of β-carotene-loaded nanoliposomes because of their lower mean size and polydispersity index, as well as better capacity inhibiting lipid peroxidation and better stability during storage.	Hamadou A.H. et al. [105]
Niosome	β-carotene	Span 40/ Span 60/ Span 80 + Tween 20/ Tween 40/ Tween 60 + cholesterol		The resulting systems showed high resistance to sunlight, high temperature, and induced oxidative stress. β-carotene was seen to be stable in culture medium up to 96 h and it was effectively taken up by cultured cells at concentrations covering the range of physiological levels (0.1–2 µM).	Palozza P. et al. [109]
Niosome	Lycopene	Span 60 + cholesterol		Lycopene showed resistance to oxidative stress. In vitro release was gradual and prolonged. Bioavailability was enhanced producing rise in blood plasma levels of 297.19%.	Sharma P.K. et al. [110]
SLN	β-carotene	Tween 80	Blends of MCT + glyceryl stearate/partially hydrogenated palm oil	SLNs prepared using glyceryl stearate were completely digested. SLNs fabricated with HPO had higher β-carotene bioaccessibility, associated with the higher amounts of monounsaturated fatty acids in the micelle fraction.	de Abreu-Martins H. H. et al. [118]
SLN	β-carotene	Whey protein isolate (WPI)	Palmitic acid + corn oil	Palmitic acid was seen to form a shell around β-carotene. The use of whey protein isolate was seen to improve the stability of SLN as well as β-carotene’s oxidative stability.	Mehrad B. et al. [119]
SLN	β-carotene/vitamin A/ω-3 fish oil	Quillaja extract/ Quillaja extract + low-melting lecithin/ Quillaja extract + high-melting lecithin		The main impact on the structural arrangement and chemical stability of the encapsulated molecules was attributed to the solubility of the functional lipids in the aqueous phase and to the crystallization temperature in relation to that of the carrier lipid.	Salminen H. et al. [120]
SLN, LLN	β-carotene	Tween 80	Cocoa butter + or/hydrogenated palm oil	LLNs had better stability to droplet aggregation, while SLN exhibited considerable increment in particle diameter. β-carotene rate degradation was seen to be higher in SLNs.	Qian C. et al. [116]
NLC	β-carotene	Tween 80/ Tween 60/ Tween 20/	Squalene + grapeseed oil + glyceryl stearate + n-hexadecyl palmitate	The smallest droplets were obtained using Tween 20 as the main surfactant. NLCs produced containing Sq and GSO were seen to enhance the antioxidant properties of the system, whereas NLCs produced with GSO and Tween 80 as the main surfactant manifested the greatest antioxidant activity towards free oxygen radicals. β-carotene-loaded NLCs revealed antibacterial activity against *Escherichia coli*, also showing a correlation with the concentration of β-carotene and of the liquid lipid, rather than the particle size.	Lacatusu I et al. [122]
NLC	Lycopene	Span 80 + Planrasens^®^ HE20	Cocoa butter + grapeseed oil	NLCs maintained lycopene’s stability when stored at 4 °C for 3 months.	Sirikhet J. et al. [123]

Note: SLN = solid lipid nanoparticle; LLN = liquid lipid nanoparticles; NLC = nanostructured lipid carrier; + = and; / = or.

## 7. Biopolymeric Nanocarriers

**Figure 4:**
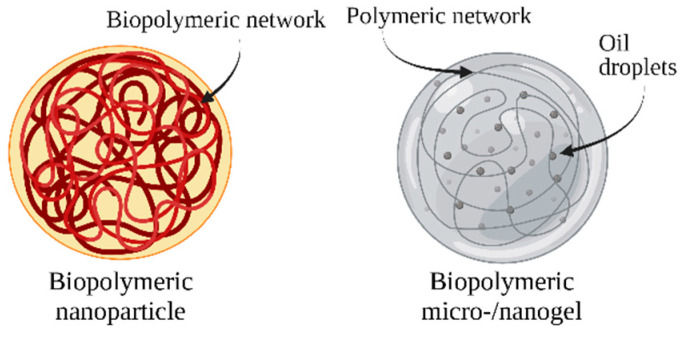
Schematic representation of biopolymeric nanocarriers.

Biopolymeric nanocarriers can be produced from various types of generally recognized as safe (GRAS) polysaccharides or proteins. A biopolymer can be used on its own or in combination with other biopolymers to create food-grade nanocarriers. The complexation of two different biopolymers allows the formation of complexed biopolymer nanoparticles, which can be formed by two different proteins, two different polysaccharides, or by a protein and a polysaccharide. When selecting a biopolymer or combination of biopolymers, the desired physiochemical and functional properties of the resulting particles, the biopolymer, and the suitability of the encapsulated bioactive compound should be considered [124,125]. The bond between a poorly soluble bioactive molecule and a polysaccharide or protein enhances its stability and protects the molecule from degradation (Figure 4) [126].

Both polysaccharide- and protein-based nanoparticles can be prepared through different techniques, including emulsification, desolvation, coacervation, and electrospray drying [127]. Below, we described in detail the main biopolymeric nanocarriers developed for carotenoid delivery, such as polysaccharide-based and protein-based nanocarriers and micro- and nanogels (Table 2).

### 7.1. Polysaccharide-Based Nanocarriers

These nanocarriers are made of polysaccharides obtained from algae (alginate and carrageenan), plants (pectin, guar gum, cellulose, inulin, starch, and maltodextrins), animals (chitosan and chondroitin), and microbes (dextran and xanthan gum). Polysaccharide-based nanocarriers are characterized by a wide diversity of reactive groups, molecular weights, and chemical compositions, and thus result in several structures with different properties [86,124,128]. Some polysaccharides can be used both in their native and modified form, depending on the use for which they are meant and the characteristics of the carried compound. For instance, water-insoluble cellulose can be used as-is or can be modified to obtain carboxymethyl cellulose (CMC), which is a more water-soluble form, or also hydroxyethyl cellulose (HEC). The latter is a suitable material to form β-carotene-loaded nanocarriers in combination with linoleic acid [86]. Similarly, starch can be enzymatically, physically (such as pre-gelatinization), or chemically (such as oxidation, esterification, etherification, partial hydrolyzation, and cross-linking) modified to acquire the desired features [84,86].

Polysaccharides are appropriate for the delivery of bioactive compounds because of their safety, biocompatibility, and biodegradability. They are also generally readily available and inexpensive [86,124,128]. In addition, polysaccharide-based nanocarriers are the most stable nano-delivery systems at high temperatures when compared to lipid- or protein-based nanocarriers, which can be melted or denatured [129]. According to their structural features, polysaccharide-based nanocarriers can be generated through different approaches, including covalent and ionic crosslinking, polyelectrolyte complexation, and self-assembly of hydrophobically modified polysaccharides [128].

In a study conducted by Arunkumar and co-workers [130], water-soluble low-molecular-weight chitosan nanocarriers were used to improve the bioavailability of lutein. Both in vitro and in vivo studies revealed a significant increase in bioavailability when the compound was encapsulated, in comparison to the non-encapsulated control.

Tachaprutinun and co-workers [131] encapsulated astaxanthin into polymeric carriers using the solvent displacement technique. The following three different polymers were evaluated: poly(ethylene oxide)-4-methoxycinnamoylphthaloyl-chitosan (PCPLC), poly(vinylalcohol-co-vinyl-4-methoxycinnamate) (PB4), and ethylcellulose (EC). The best results were obtained by using PCPLC, which had a high encapsulation (98%) and loading (40%) efficiency. Furthermore, astaxanthin-loaded PCPLC was highly stable when treated with heat at 70 °C for 2 h in an aqueous environment. In contrast, EC was unable to encapsulate astaxanthin, PB4 had a poor encapsulation efficiency, and both were completely degraded by heating.

Rutz and co-workers [132] encapsulated β-carotene and palm oil into chitosan/sodium tripolyphosphate or chitosan/carboxymethylcellulose carriers to evaluate their tunability with food systems (yogurt and bread) and examined their release profile using a simulated digestive model. In this case, the nano-structuration process of the biopolymers resulted in the formation of microparticles. The chitosan/carboxymethylcellulose-loaded carrier presented an optimal release behavior in aqueous media and gastric fluid. However, the release percentage in the intestinal fluid was low, even if it increased when included in food systems. These carriers were also observed to have a lower β-carotene release rate during storage. In contrast, chitosan/sodium tripolyphosphate carriers presented good β-carotene release in aqueous media and gastric fluid and acceptable release in intestinal fluids. Similar to its counterpart, β-carotene released from these carriers was enhanced when included in food systems. On the other hand, these carriers tended to release a higher quantity of β-carotene during storage.

### 7.2. Protein-Based Nanocarriers

Proteins for the fabrication of nanocarriers are normally obtained from animals (whey proteins, caseins, gelatin, collagen, albumin, elastin, and silk), plants (soy proteins, cereal proteins, zein, gliadin, and pulse proteins), or microorganisms. Proteins can be used as they are or can be chemically, physically, or enzymatically altered to modify their functionality according to the desired result [86]. Proteins are easily digested in the human gastrointestinal tract and can successfully release the encapsulated compound during digestion. Additionally, proteins frequently present antioxidant properties, which are useful in the preservation of the carried bioactive [124].

For example, casein micelles are broadly used to encapsulate lipophilic molecules. Sometimes, a self-assembly approach is also pursued to create artificial micelles called reassembled casein micelles, using sodium caseinate or isolated caseins as the starting material.

Sáiz-Abajo and co-workers [133] used the self-assembling approach to encapsulate β-carotene, thus obtaining loaded, reassembled casein micelles. The authors demonstrated that these nanostructures had a protective effect against degradation processes that would otherwise naturally occur during industrial treatments, such as sterilization, pasteurization, high hydrostatic pressure, and baking.

Further studies reported the use of native casein micelles and β-casein micelles as suitable carriers for lipophilic compounds [134]. For instance, in a study conducted by Moeller and co-workers [135], native casein micelles were used as nanocarriers for β-carotene; the micelles were primed at pH 5.5, 2 °C, for 5 min and successively loaded with β-carotene.

Chen and co-workers [136] investigated the effects of β-carotene encapsulation on ferritin nanocages. The β-carotene-ferritin composites were highly water soluble, and the thermal stability of β-carotene was noticeably improved.

Pérez-Masiá and co-workers [137] observed that a higher encapsulation efficiency was obtained when lycopene, previously dissolved in soybean oil, was encapsulated using a whey protein concentrate in comparison to carbohydrate-based matrices, such as dextran or chitosan. Whey protein capsules also presented better protection against moisture and thermal degradation. Different methods were used, such as electro spraying, spray drying, and coaxial electro spraying. Spray drying was observed to affect lycopene stability and encapsulation efficiency due to the high temperatures required for the process.

Cheng and co-workers [138] prepared lutein-loaded zein nanoparticles through antisolvent nanoprecipitation and investigated their stability by using an in vitro gastrointestinal model. While the stability was enhanced by 58%, micellization efficiency decreased by 42% because of the tendency of zein nanoparticles to aggregate and precipitate in the presence of the salts used to recreate physiological conditions. Nanoparticles were not completely digested by gastric enzymes at high ion concentrations. This study suggested that zein nanoparticles may be useful to prevent the degradation caused by gastric conditions; however, their application might be counterproductive for the loaded compound’s bioaccessibility.

### 7.3. Biopolymeric Microgels and Nanogels

Microgels and nanogels (Figure 4) consist of dispersions of hydrogel microparticles or nanoparticles, which are formed by three-dimensional polymeric networks (usually proteins or polysaccharides) created from physical or chemical crosslinking. These systems can hold high volumes of liquid [86,139].

These systems are formed when a porous matrix that can entrap oil droplets within itself is then dispersed in an aqueous medium, resulting in an O/W1/W2 nano-emulsion [140]. Furthermore, the affinity of hydrogels to absorb water is defined by the presence of hydrophilic groups (such as -OH, -CONH, -CONH_2_, and -SO_3_H) within the polymers forming hydrogel structures. A hydrogel can be hydrated at different degrees according to the number of these hydrophilic groups. However, a much lower hydration capacity is achieved if hydrophobic polymers are used [141].

Nano- and microgels are suitable for the delivery of both lipophilic and hydrophilic molecules. Lipophilic molecules are firstly dissolved in an O/W emulsion to obtain oil droplets and are then combined with the biopolymer solution. Nano- and microgels can be fabricated using different methods, such as extrusion, phase separation, antisolvent precipitation, and templating methods. Microgel dimensions range between 100 nm and 1000 µm, while nanogel dimensions were <100 nm [88]. Microgels and nanogels are generally opaque due to both the size of the oil droplets contained in the hydrogel particles (in the order of the wavelength of light) and because of the hydrogel particles themselves (on which light scattering occurs). Furthermore, both the lipid droplets within the hydrogel system and the nanogel/microgel itself tend to have the same instability problems that occur in conventional emulsions, such as gravitational separation, flocculation, and coalescence. On the other hand, it is easier to control the release rate of bioactive molecules from hydrogels than in emulsions due to the possibility to modulate the composition or dimension of the particles. For instance, the release of lipophilic droplets can be slowed down by increasing the entanglement of the hydrogel network in which they are contained. This causes a delayed digestion rate because of the increased diffusion path length that the lipase must cover to reach the droplets. Another important factor that influences the rate of droplet digestion is the permeability of the hydrogel particles, which can be modulated by varying the formulation ingredients. In particular, the rate of digestion is reduced as the hydrogel permeability also decreases [83].

Nano- and microgels can be prepared using various polymeric materials, including collagen, albumin, fibrin, chitosan, hyaluronic acid, heparin, chondroitin sulfate, agarose, and alginate [139]. Zhang and co-workers [142] investigated the effects of the encapsulation of β-carotene in alginate-based hydrogel beads. The latter was fabricated by injecting a mixture of alginate molecules and β-carotene-loaded lipid droplets into a calcium ion solution using an extrusion device. The following delivery systems were considered: free lipid droplets (nanoemulsion) and loaded hydrogel beads formed using either 0.5% or 1% alginate. β-Carotene encapsulated into alginate-based hydrogel beads was observed to be generally much more stable to chemical degradation if subjected to high temperatures and storage when compared to β-carotene-loaded nanoemulsions. Hydrogels formed using 1% alginate provided the best protection against degradation. On the other hand, simulated gastrointestinal studies revealed that β-carotene-loaded nanoemulsions were more bioaccessible than their counterparts. In particular, the bioaccessibility was lower when the hydrogel beads contained a higher amount of alginate; this effect can be attributed to the entrapment of some β-carotene in undigested lipid droplets contained in hydrogels.

**Table 2:** Summary of different studies testing the encapsulation of carotenoids in polymer-based nanocarriers.

Delivery System	Loaded Compound	Biopolymer	Study Outcomes	Reference
Polysaccharide-based nanocarrier	Lutein	Chitosan	Lutein bioavailability was enhanced by 27.7%. Moreover, postprandial lutein levels in blood plasma (54.5%), liver (53.9%), and eyes (62.8%) in mice were much higher than the control.	Arunkumar R. et al. [130]
Polysaccharide-based nanocarrier	Astaxanthin	Poly(ethylene oxide)-4-methoxycinnamoylphthaloyl-chitosan (PCPLC)/ poly(vinylalcohol-co-vinyl-4-methoxycinnamate) (PB4)/ ethylcellulose (EC)	Encapsulation into PCPLC showed the best results, with high encapsulation efficiency (98%), loading (40%), and high stability to heat. On the contrary, encapsulation into PB4 and EC did not produce positive results.	Tachaprutinun A. et al. [131]
Polysaccharide-based nanocarrier	β-carotene	Chitosan + sodium tripolyphosphate/ chitosan + carboxymethylcellulose	The chitosan and sodium tripolyphosphate carrier showed considerable β-carotene release in aqueous media and gastric fluid, and adequate release in intestinal fluids. The chitosan and carboxymethylcellulose carrier showed an optimal release behavior in aqueous media and gastric fluid; however, the release percentage in the intestinal fluid was small. In both cases, β-carotene release was enhanced when included in food systems.	Rutz J. et al. [132]
Protein-based nanocarrier	β-carotene	Casein	The nanocarrier successfully protected β-carotene during sterilization, pasteurization, high hydrostatic pressure, and baking.	Sáiz-Abajo M.J. et al. [133]
Protein-based nanocarrier	β-carotene	Native β-casein	Micelles were optimally primed at pH 5.5 with a temperature of 2 °C for 5 min, and successfully loaded with β-carotene.	Moeller H. et al. [135]
Protein-based nanocarrier	β-carotene	Ferritin	The resulting nanocages became highly water-soluble and the thermal stability of β-carotene was improved.	Chen L. et al. [136]
Protein-based nanocarrier	Lycopene (dissolved in soybean oil)	Whey protein/ carbohydrate-based matrices (dextran/chitosan)	The whey protein nanocarrier showed a higher encapsulation efficiency compared with the carbohydrate-based ones, as well as better protection against moisture and thermal degradation.	Pérez-Masiá et al. [137]
Protein-based nanocarrier	Lutein	Zein	The stability was tested in an in vitro gastrointestinal model, and it was seen to be enhanced by 58%; however, micellization efficiency decreased by 42%.	Cheng C. J. et al. [138]
Hydrogel beads	β-carotene	Alginate	β-carotene was encapsulated into hydrogel beads formed by using 0.5% alginate, 1% alginate, or into nanoemulsions. The hydrogel beads were generally seen to better prevent the compound from chemical degradation, in particular hydrogels with 1% alginate provided the best protection. However, gastrointestinal studies showed that nanoemulsions were more accessible than the hydrogel beads.	Zhang Z. et al. [142]

Note: + = and; / = or.

## 8. Conclusions

In the current review, several nanostructured systems were described as able to encapsulate and deliver carotenoids. These systems were made of different materials, including lipids, polysaccharides, and proteins, and presented different degrees of loading and delivery efficiency. They were also effective in enhancing the bioaccessibility and bioavailability of the enclosed compounds while preserving their stability to oxidation and thermal degradation. The wide variety of available formulations allowed us to choose the system that best fit the chemical characteristics of the food matrix and its processing operations, thus in principle allowing the ability to enrich any type of foodstuff with compounds of nutritional relevance. However, without prejudice to their clear technological values, the safety of these nanostructured delivery systems is yet to be fully understood, and a thorough quali–quantitative evaluation of the risks arising from their intake is highly desirable.

As for the nanostructured systems, the risk assessment paradigm used, to date, for chemicals is certainly applicable, but it is not sufficient to provide a definite safety profile. Indeed, a careful assessment of the stability of these systems is also important, as their specific biological effects are inherently linked to the maintenance of nanomolar dimensions. Once ingested, nanoparticles may considerably change while passing along the complex physiological environment of the gastrointestinal tract. Furthermore, they may modify the size, structure, composition, interfacial properties, and physical state, thus altering their metabolic fate and safety profile. Materials maintaining a nanometer scale may exert local adverse effects in the gastrointestinal tract. Moreover, they can penetrate the biological barrier of the intestine and enter the circulatory system, thus posing immunological and toxicological risks [143]. On the contrary, when the nanostructured systems completely dissolve, releasing the loaded compounds, there is no reason to believe that they behave differently to the non-nanochemicals they are made of. In any event, the metabolic fate of the nanostructured carrier affects the bioavailability of the loaded nutrient; thus, it must be thoroughly investigated.

There is still little evidence regarding the short-term toxicity associated with the administration of biodegradable nano-delivery systems for bioactive compounds. In vitro studies are often insufficient to assess the potential toxicity of nanoparticles to humans; therefore, they should be studied in vivo to obtain more reliable results. However, due to ethical issues for the utilization of animal models in long-term studies and the difficulties in performing long-term studies on humans, the identification of the potential risks associated with the ingestion of nanoparticles still remains challenging. Once safety has been confirmed, also in relation to the consumed dose, these nanostructured systems will provide a simple and effective means to enrich food matrices for health purposes.
